# Health Tract No. 343

**Published:** 1868-07

**Authors:** 


HEALTH TRACT No. 343.

### VENTILATION

Is keeping the air of a room as near as possible to the purity of the external atmosphere ; the chief contaminating ingredient of indoor air is carbonic acid gas, which is poured from the lungs at every expiration, each breath intensifying the impurity, and being loaded with watery vapor, it is heavier than the common air and falls to the floor ; and if openings were there made for the egress of this bad, heavy air, but somewhat warmed, so that it could be made to pass to the flue in contact with the under surface of the floor, it would aid in keeping it warm, where warmth is more needed than at any other part of the room ; but by present modes of heating, the air at the ceiling is ten or fifteen degrees warmer, and if ventilators are put there, the warmest and purest air of the apartment escapes ; but if the bad, colder and heavier air is let out through the floor, and pure warm air is let in at the ceiling or transoms over the doors, it naturally follows after, descending to the floor in turn, as it becomes colder, more impure and heavier, and in its turn, would pass out as heavy bad air. This being so, heat would be economized, if stoves or furnaces were placed in the halls, the stairways cased up and the warm air sent into the rooms through the open hall doors or their transoms. If the dryness of the air causes uncomfortable sensations, moisture could be communicated by passing an iron tube through the stove where the fuel is, and arranging to have water flowing through it in such a manner as to give a surface of boiling water, sufficiently extensive to prevent any uncomfortable sensations ; it might answer every purpose to have a broad porcelain dish on the stove, to be washed and replenished with pure water several times a day ; and in addition, when the room is vacated in going to meals, or there are other absences, even for a few minutes at a time, a window and an opposite door might be opened.

